# “Look Before You Leap”: Robotic Resection of a Jejunal Mesenteric Pseudocyst

**DOI:** 10.7759/cureus.4750

**Published:** 2019-05-24

**Authors:** Raguraj Chandradevan, Ian Rutkofsky, Kristal Sirju, Felisha L Kitchen, John T Williams

**Affiliations:** 1 Internal Medicine, Coliseum Medical Centers, Macon, USA; 2 Psychiatry, California Institute of Behavioral Neurosciences and Psychology, Fairfield, USA; 3 Obstetrics and Gynecology, Coliseum Medical Centers, Macon, USA; 4 General Surgery, Coliseum Medical Centers, Macon, USA

**Keywords:** mesenteric cyst, robotic resection

## Abstract

A rare case of a jejunal mesenteric pseudocyst treated by robotic resection is reported. A 25-year-old woman was admitted to our hospital with intermittent abdominal discomfort which was exacerbated by strenuous physical activities. Physical examination revealed a fluctuant mass without tenderness. Contrast-enhanced computed tomography revealed a 4 cm-sized non-enhancing heterogeneous mass on a proximal small bowel loop mesentery. Based on the findings, a differential diagnosis of a gastrointestinal stromal tumor, hematoma, desmoid tumor, and mesenteric cyst was made. Robotic diagnostic laparoscopy was performed to obtain an accurate diagnosis and treatment. Exploration of the cavity revealed a 4 cm fairly mobile mass originating from the mesentery of the jejunum. Segmental resection of the jejunum and its mesentery, including the mass and extracorporeal anastomosis, was performed without any complications. Macroscopically, the mass was cystic and the lumen had grumous material. The final pathological diagnosis was a mesenteric pseudocyst. The patient had an uneventful postoperative course.

## Introduction

Mesenteric cysts are very rare abdominal growths. They may be localized anywhere in the mesentery, from the duodenum to the rectum, but they never have been reported to originate from or be attached to any abdominal organs. In clinical presentations, these cysts are most commonly located in the small bowel mesentery (50% - 80%). The second most common location is in the large bowel mesentery (15% - 30%), and very rarely, they can present in the retroperitoneal space (7% - 20%) [1]. The histological classification and imaging correlation of these cysts were clearly differentiated in 1987, and Ros et al. used the term “non-pancreatic pseudocyst“ for the first time [2]. Based on the histopathological features, these formations can be classified into six groups, including cysts of lymphatic origin--lymphatic (hilar cysts) and lymphangiomas, cysts of mesothelial origin--benign or malignant mesothelial cysts, enteric cysts, cysts of urothelial origin, dermoid cysts, and pseudocysts--infectious or traumatic etiology [3]. The preoperative diagnosis of mesenteric pseudocyst is usually difficult because of the lack of disease-specific signs [4]. Clinicians should suspect a mesenteric mass in the presence of painful abdominal pressure with normal laboratory findings. In symptomatic cases, acute or chronic abdominal pain is the most common feature. Presentation is highly varied, depending on the localization, size, and interference of abdominal organ compression. This can especially cause intestinal obstruction, hydronephrosis, and lower extremity edema. Diagnostic aids include abdominal computed tomography, abdominal ultrasound, upper gastrointestinal (GI) tract series, barium enema, and intravenous pyelogram. These can assist in excluding GI or genitourinary cysts, tumors, or any organ compression. Accurate diagnosis prior to surgery is difficult because the origin of the lesion and internal aspects of the cyst cannot be determined precisely by radiological examination [5]. Treatment of choice is resection/enucleation; resection of the adjacent bowel may occasionally be necessary. Morbidity, mortality, and complications of the operative procedures are kept low due to emerging advanced surgical techniques and follow-up procedures. Most of these patients undergo a minimally invasive procedure and recover within a few days. To our knowledge, about 20 cases of (or with) jejunal mesenteric cysts have been reported in the English literature [4]. We report our experience with a young patient who was diagnosed as having a mesenteric (non-pancreatic) mass that originated from the mesentery of the jejunum. As a result, a diagnostic laparoscopy was performed with robotic resection as treatment.

## Case presentation

A 25-year-old young woman was admitted to our hospital with intermittent periumbilical abdominal discomfort and pain. She had a history of depression and was otherwise a healthy individual. She complained that the discomfort was ongoing for six to eight months, and she usually rated it three to four out of 10 on the pain scale. There was no association with her menstrual cycle or food nor was there any associated nausea, vomiting, constipation, or diarrhea. There were no alleviating factors but she reported exacerbated discomfort with a strenuous workout at her school. The pain and discomfort were infrequent and usually occurred five to six times per month with a duration of one to two hours of constant brief episodes. It usually resolved spontaneously, and for this reason, she did not pay it much concern. There was no increase in frequency or intensity of the discomfort. She never noticed a fever nor any distention, bloating, or dyspepsia. There was no history of known abdominal trauma. She had no family record of any similar symptoms or history. Vital signs, including blood pressure, heart rate, respiratory rate, and body temperature, were all in normal ranges. However, while the physical examination was negative for tenderness, it revealed a non-specific mass of 5 cm x 3 cm that was palpable in the left upper quadrant. The mass seemed to arise from a deep origin below the abdominal wall; it had a smooth surface without any tenderness. It was freely mobile below the abdominal wall and appeared fluctuant. Blood test results found that the hemoglobin level was 11.6 g/dL (normal range 12 g/dl - 15.5 g/dl), the white blood cell was 10.5 K/µL (normal range 4 /lµ - 11 /µl), the platelet count was 149 K/µL (normal range: 150 K/lµ - 450 K/µl), the erythrocyte sedimentation rate was 5 mm/h (normal range: 0 mm/h - 29 mm/h), and the C-reactive protein level was 2 mg/L (normal range below 3.0 mg/L). Liver function tests, basic metabolic panel, amylase and lipase levels, and urinalysis were all within normal limits.

**Figure 1:**
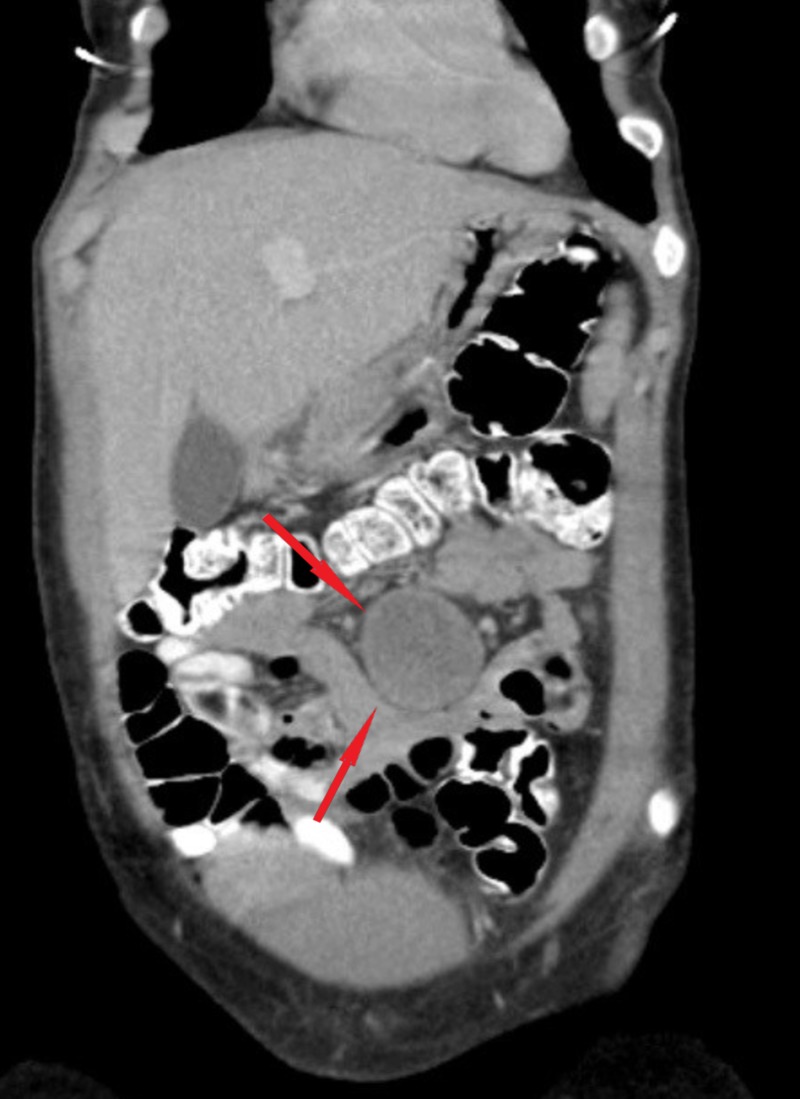
Coronal view of computed tomography scan of the abdomen showing the mesenteric cyst (red arrows)

**Figure 2:**
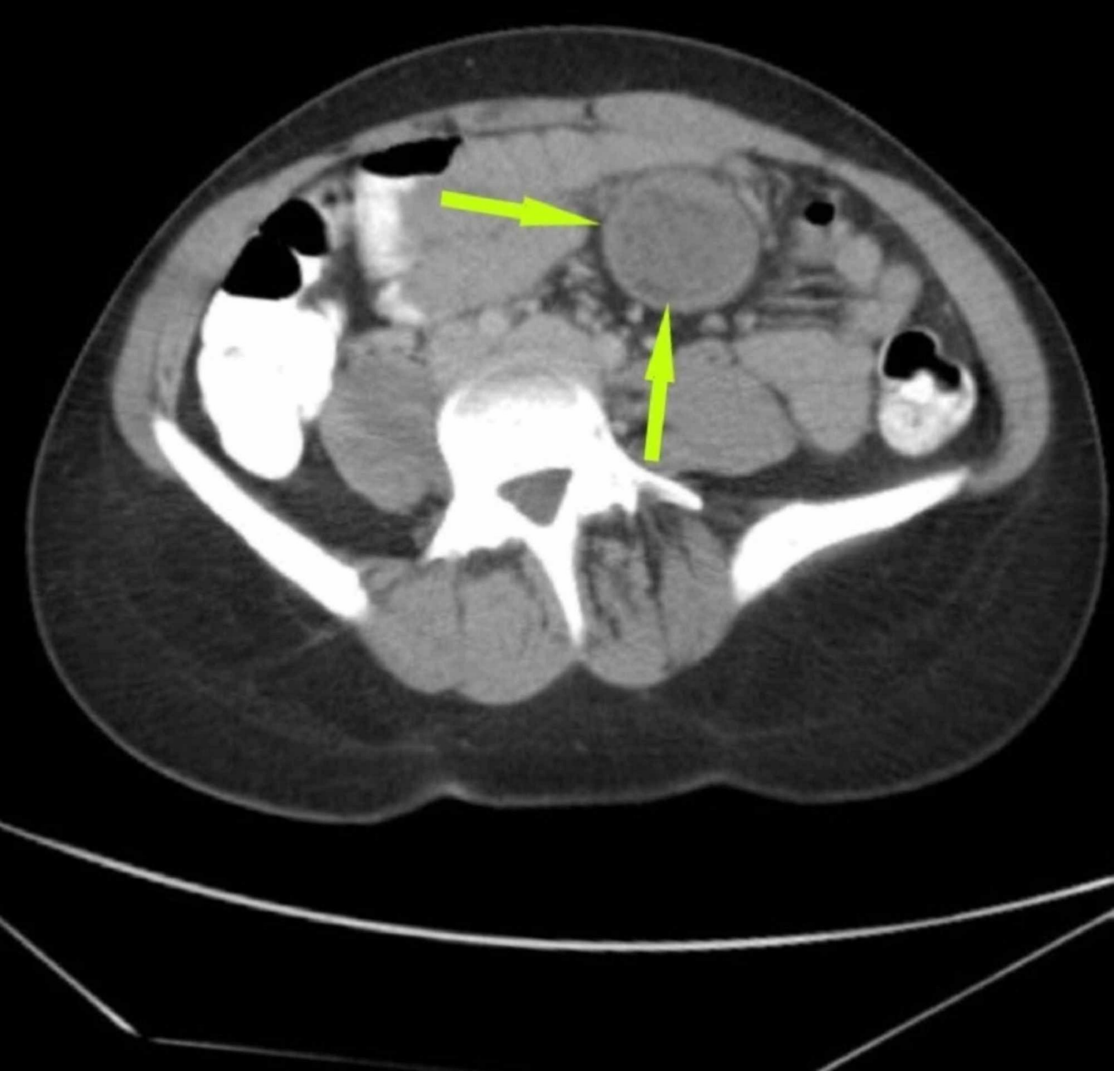
Computed tomography scan of the abdomen showing the mesenteric cyst (yellow arrows)

Contrast-enhanced computed tomography (CT) revealed a non-enhancing intra-abdominal mass (4.2 x 4.3 cm) with a heterogeneous pattern located on a proximal small bowel loop at the mesenteric border (Figures 1-2). CT findings were not supportive to distinguish whether the mass was separated or attached to neighboring vessels or organs. Based on the history, physical examination, and imaging studies, a differential diagnosis of a gastrointestinal mesenteric tumor, stromal tumor, mesenteric hematoma, dermoid cyst, or desmoid tumor were made. Laparoscopy was performed to obtain an accurate visual diagnosis.

**Figure 3:**
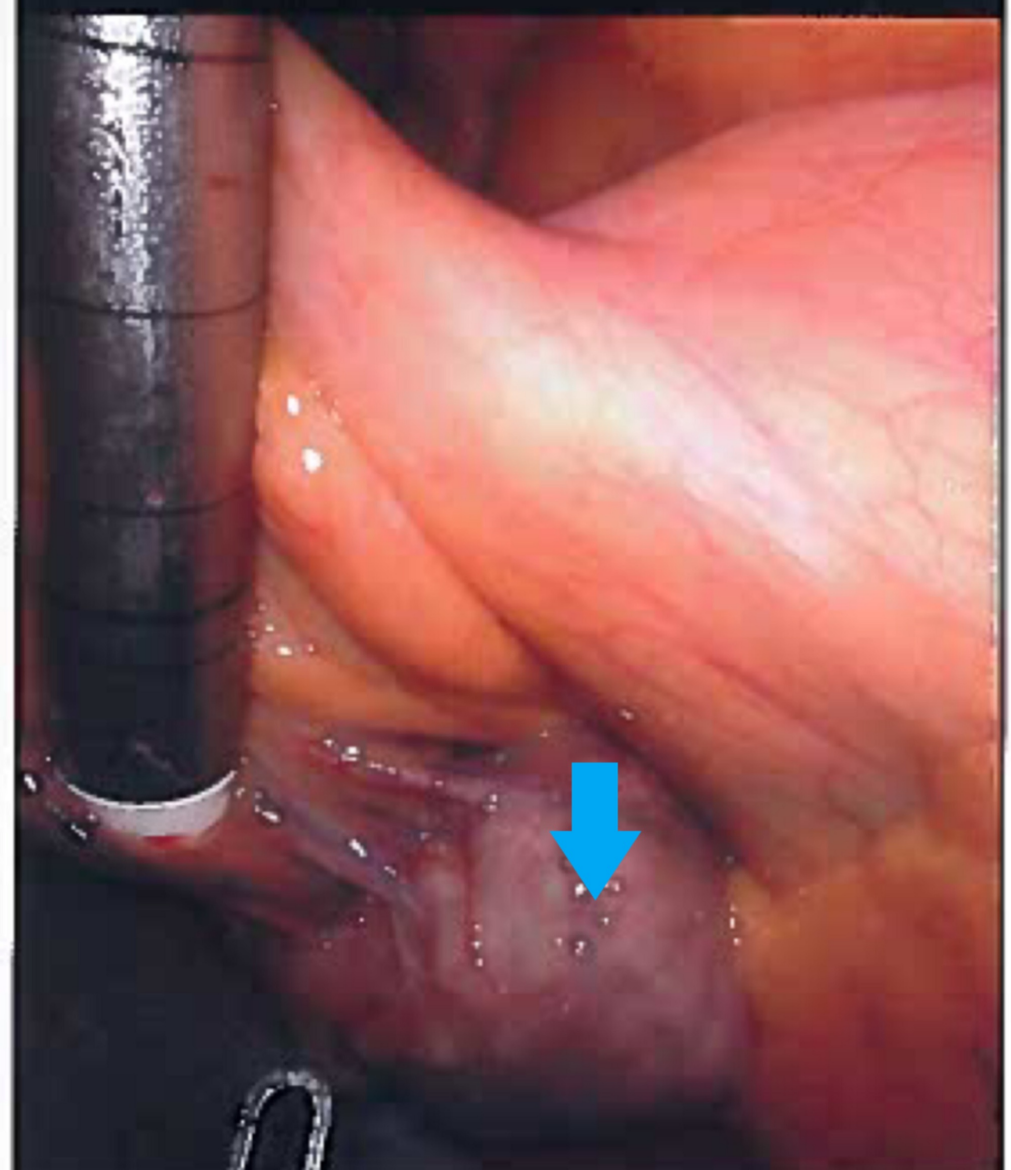
Robotic camera view of the mesenteric cyst (before clamping)

**Figure 4:**
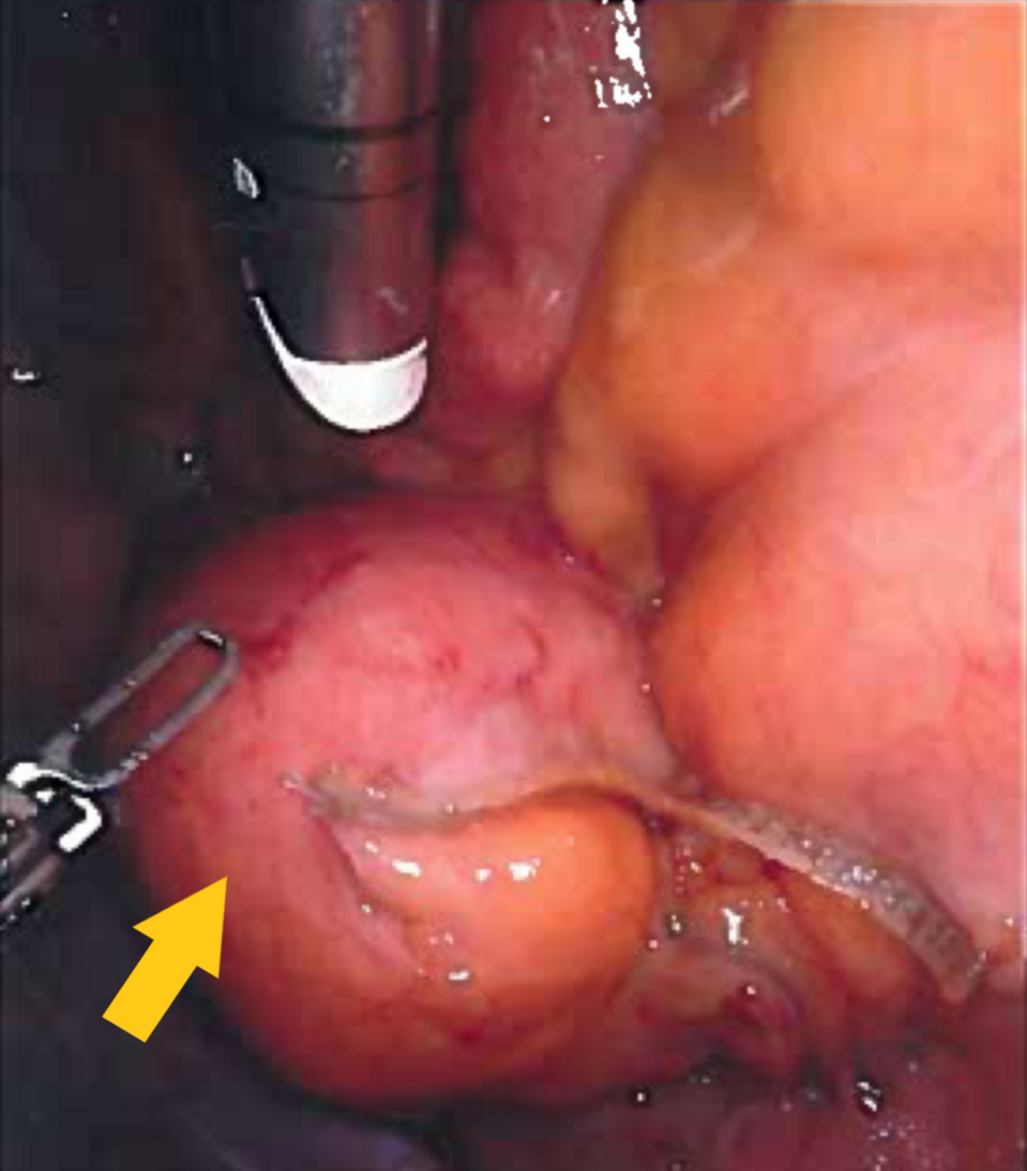
Robotic camera view of the mesenteric cyst (with stapler clamp)

Exploration of the abdominal cavity identified a 4 cm mass (Figure 3), which originated from the mesentery of the jejunum. The mesentery was shortened because of the location and size of the mass. Segmental resection of the jejunum and its mesentery, including the mass, was performed via a Da Vinci robotic system (Figure 4). The procedure was completed with extracorporeal anastomosis of the jejunal segment. Macroscopically, the mass appeared to be a cystic mass of the jejunal mesentery and it measured 4.2 cm x 4.4 cm. The mass was cystic and the walls were pink-tan. The lumen of the cyst was dull trabeculae and demonstrated unremarkable rugal folds without mass lesions. It consisted of large amounts of grumous material and the overall change was degenerative in nature. Culture of the contents were sterile for bacteria, fungal, or tuberculous material. Histopathological examination of the resected tissues revealed a cystic wall that was made up of dense fibrotic tissue with scattered chronic inflammatory cells and lymphoid aggregates. The lesion did not show any significant epithelial lining with focal histiocytic or specific epithelium nor was there any proliferating/dysplastic lining. The final pathological diagnosis was (non-pancreatic) inflammatory pseudocyst. The patient had an uneventful postoperative course. We followed up with the patient in two weeks and again in three months. During the follow-up, the patient reported that removal of the cyst helped her abdominal pain and she improved clinically.

## Discussion

Mesenteric cysts are rare intra-abdominal lesions arising with an incidence of one in 100,000 admissions of adults and one of 20,000 admissions of children [6]. Mesenteric cysts originate from the mesentery and have no connection with retroperitoneal organs; they are mobile and can present in any part of the mesentery. In our case, the presentation of the cyst is from the jejunal mesenteric, and according to prior reports, they occur most frequently in the small bowel mesentery with an incidence rate of 50% - 80%. Although these benign cysts are classified with embryological and causal standards, they are divided into six groups according to the histological features [7]. According to these classifications, our patient suffered from a non-pancreatic pseudocyst. These non-pancreatic pseudocysts are histologically similar to pancreatic pseudocysts, and the wall of the cyst is composed of fibrous tissue and characterized by the absence of epithelial lining cells.

Very few cases have been reported in the past regarding mesentery pseudocysts [8]. Most of such lesions occur secondary to trauma or infection [9], but in the present case, our patient did not have a history of abdominal trauma or abdominal inflammatory disease. As most cysts have nonspecific symptoms, they are often diagnosed incidentally, either by ultrasonography or CT. It is difficult to detect them on clinical examination or by laboratory markers. Initial clinical presentation includes vague abdominal pain, vomiting, constipation, and a palpable abdominal mass [3]. As in our case presentation, the patient presented with abdominal discomfort and pain, which is the most common manifestation (80%). Late complications, such as torsion, rupture, hemorrhage, infection, and intestinal obstruction, have been described [10]. On CT images, these cysts are hypodense, have thin walls, and show no enhancement with contrast. In our presentation, the CT showed a non-enhancing mass with a heterogeneous pattern, and the cyst was filled with grumous material on pathological analysis. Such content is unique to a pseudocyst. 

Robotically-assisted surgeries have become more commonly used in a variety of surgical procedures. Improved precision and dexterity with “wristed instrumentation” allows for freedom to maneuver all laparoscopic incision sites comfortably. Additionally, a three-dimensional image allows for an improved ability to visualize the precise anatomical location of interest with reduced fatigue, further minimizing fatigue-related complications such as tremor. The availability of the robot and the robotic stapler allowed us to provide the benefit of minimally invasive surgery. In addition, robotic procedures can reduce surgical time, thus lowering the rate of postoperative complications, minimizing exposure of anesthetic agents, and reducing the recovery time postoperatively [11]. In our case, the mesentery was shortened because of the size and location of the cyst. We considered marsupialization, but because of the recurrence risk, we opted an alternative approach - robotic resection. As a result, we performed an extracorporeal anastomosis using a robotic stapler, which helped us to deliver the cyst and anastomosing site through the focused incision. The robot’s aid substantially benefited our visual field and the complexity of the extraction, especially considering the presence of a shortened mesentery.

## Conclusions

Although mesenteric pseudocysts are rare and preoperative diagnosis is difficult due to non-specific signs and symptoms, the disease must be considered in the differential diagnosis of intra-abdominal masses. They can be successfully managed by complete resection; laparoscopic and robotic excision of these cysts have become an increasingly popular option. We believe that robotic resection might serve as an alternative treatment for mesenteric cysts when the cysts are of small to moderate size and, more specifically, when the patient has a shortened mesentery. Surgical alternatives to the midline or paramedian incision, such as laparoscopic and further advanced robotic mesenteric cyst excision, warrant further data to determine if morbidity is significantly reduced. In this presentation, we have shown that robotic mesenteric cyst excision and bowel anastomosis is a safe alternative to open surgical enucleation.
